# The developmental pathways of senior international soccer players: A 13-year analysis of the career trajectories of the Swedish men’s senior international team

**DOI:** 10.1371/journal.pone.0316216

**Published:** 2025-03-04

**Authors:** Liam Sweeney, Anton Kalén, Andreas Ivarsson, Tommy R. Lundberg

**Affiliations:** 1 Department of Sport Science and Nutrition, Faculty of Science and Engineering, Maynooth University, Kildare, Ireland; 2 Swedish Olympic Committee, Stockholm, Sweden; 3 School of Informatics, University of Skövde, Skövde, Sweden; 4 Department of Health and Sport, School of Health and Welfare, Halmstad University, Halmstad, Sweden; 5 Department of Sport Science and Physical Education, University of Agder, Agder, Norway; 6 Department of Laboratory Medicine, Division of Clinical Physiology, Karolinska Institutet, Stockholm, Sweden; 7 Unit of Clinical Physiology, Karolinska University Hospital, Stockholm, Sweden; Universidade Federal de Goias, BRAZIL

## Abstract

This study explored the developmental pathways of all players (n = 313) who represented the Swedish men’s senior international team between 2011 and 2023 (n = 118) and/or the U21 international team between 2011–2022. We also examined at which respective level each player’s youth club was ranked (i.e., premier, second, or third division club, or international academy) and the age at which they were first present in that club environment. Of the 118 senior international players, 34% were selected at U15–U16, 33% were selected at U17–U18, and 33% were selected at U21 or the senior international level. Later selected (U21 and senior) players had a later senior international debut than early selected (U15–U16) players (-2.5 years, 95% CI [-4.0, -1.0 years]). Later selected players also made their senior club debut later than those selected at the U17–U18 (-1.3 years, 95% CI [-2.0, -0.5 years]) and U15–U16 (-1.9 years, 95% CI [-2.6, -1.1 years]) international level. While the majority (60%) of senior international team players entered a premier division club at some point during their junior years, players from lower clubs were overrepresented among the players who reached the senior international team without previous international team experience and made a later debut in the senior international team. We conclude that senior international players have different career trajectories and that this should be accommodated by providing structures that allow players to progress into, and out of, different development environments that best suit their individual needs as they progress to the senior level.

## Introduction

To develop youth athletes with the potential to reach the elite senior levels of competition, across sports, National Federations have implemented systematic talent identification strategies and formalised talent development (TD) programmes that span local, regional, national and international levels [1,2]. Such TD programmes are seen as an essential ‘tool’ to promote the development of the highest potential youth athletes, inciting the investment of significant resources (e.g., financial, time, logistics, personnel) into such programmes [3]. Those youth athletes selected into such programmes are typically exposed to increased developmental provision (e.g., increased training volumes and contact hours, access to highly qualified coaches, performance analysis) relative to those not selected in an attempt to facilitate long-term development [3].

One particular sport that has seen a significant (and ever increasing) strategic investment in such TD programmes in recent decades is soccer (herein referred to as football) [4]. In the United Kingdom, for example, thousands of male players are selected each year into the academies of professional football clubs, with initial player selection beginning from as young as age five years in some contexts [5,6]. Despite this approach, the effectiveness of early selection has consistently been questioned across the literature, especially given the dynamic, unpredictable, and non-linear nature of TD [3,7,8]. Consequently, there is significant debate regarding the efficiency of such TD programmes, particularly at the national and international level [9]. For example, average annual turnover rates of 41% have been reported at the junior international level in Germany [10]. Within the same Association, between 1987 and 1994, 73% of those selected for the German U16–U21 international squads were subsequently deselected [11]. Similarly, a report based on data from the Italian Football Association [12] showed that < 20% of senior international players represent Italy at the Under 16 level.

In response to these findings, there have been suggestions to delay the age at which players are selected into such programmes and to widen developmental opportunities to broader populations [1,13]. Given that the most successful senior athletes do not achieve an equivalent competition as junior athletes [8,14], there have been significant questions raised regarding the purpose of formalised youth TD programmes. In this regard, several authors have argued that the significant resources invested into such programmes are likely better spent when invested in a broader population at the community level with a focus on recruitment, participation and development, rather than invested in a very select minority [8,13,15], the vast majority of which will not become elite senior players [3,8,14,16–20].

Research conducted in alternate contexts has, however, shown contrasting associations between early selection and senior participation in professional and international football. Söderström and Garn [21] note that players who were selected into the Swedish Football Association’s U15 regional TD programmes were twice as likely to play professional football than those who were not. In an examination of the pathways of over 9500 European youth and senior international footballers, only a quarter of senior international players from England, France, Italy, Germany, and Spain between 2002–2022 did not represent their country at the junior international level [22]. Furthermore, nearly one third of professional footballers in the top two tiers in German club football between 2009–2012 had made an appearance for their junior international team [10] and over 60% of Portuguese junior international players selected aged 17–18 years made a senior international appearance [23]. Thus, whilst youth to senior transitions are generally low, exposure to the significant developmental resources, experiences, and challenges associated with academies and junior international teams can be a prominent feature in the development of some senior professional footballers.

It is important to recognise that the developmental pathways of high potential young players span beyond their junior international environments [24]. The broader context and how other developmental environments may simultaneously influence players’ developmental trajectories should therefore be explored. In this regard, there have been recent calls for greater longitudinal, multimethod, and contextually situated studies in TD that track individual athlete pathways [25]. This requires not just an examination of players’ junior international experience, but also their multiple club environments and the relative ‘professionalisation’ of these environments [4].

To address this knowledge gap, in this retrospective study we examined the developmental pathways of all players who represented the Swedish men’s senior international team between 2011 and 2023. Specifically, we were interested in exploring when/if players were first selected for a youth international team and in which respective age groups they made appearances prior to making a senior international appearance. We also sought to examine at which age each player made their first appearance at the senior international level. Thirdly, we examined at which respective level each player’s youth club was ranked (i.e., premier, second, or third division club, or international academy) and the age at which they entered this club environment. Given the non-linear and complex nature of TD [7], it was hypothesised that the senior international players would have varied career trajectories, with a spread of junior international experience across age groups. We also hypothesized that the highest ranked clubs would provide the most senior international players.

## Methods

### Study design

The Swedish Football Association is the National Governing Body for football in Sweden. At the developmental level, the Swedish Football Association implement youth international teams for male players at the U15, U16, U17, U18, U19, and U21 level. As part of the Swedish Football Association’s male international pathway, those players considered the highest performing in the country within their respective age group are selected into each youth international team. The players selected into these youth international squads are exposed to additional training, coaching, sport science, medical support, and international travel and competition in an attempt to support their development to the senior international level. Subsequent to the U21 international team is the senior international team.

At the club level in Sweden, many of the formal football clubs have implemented their own academy. The formal clubs in Sweden are ranked by a tiered (pyramid) system, reflective of the league that the senior/first team competes in on a national scale. The tiered club system in Sweden in order of highest to lowest ranked clubs are as follows: Premier division clubs (i.e., Allsvenskan; the highest professional football league in Sweden), first division clubs (i.e., Superettan), and second division clubs (i.e., Ettan). There are no formalised age requirements for selection into formal clubs in Sweden, although selection into the ‘football schools’ of Allsvenskan/premier division clubs can begin as young as age four years. Given the European Ruling on international player transfers [26], Swedish players, from age 16 years, can also be recruited into the academies of professional football clubs outside of Sweden, so long as those nations are in the European Union.

### Participants

The current study focused on all male players who were selected for the Swedish Men’s international team between 2011–2023 and/or the U21 international team between 2011–2022. The extra year of analysis for the senior team relative to the U21 team was decided so that those players in the U21 team in 2022 who were perceived as the highest performing could be selected for the senior team within the following 12 months, and subsequently included in this study. This led to a total of 313 players, 118 of which made an appearance for the senior international team, excluding players who had only participated in friendly (non-competitive) fixtures. Thereafter, our sample was divided into three groups: a) players who were selected into the senior international team *and* the U21 international team, b) players who were selected for the U21 international team, but not the senior international team, and c) players who were selected for the senior international team, but not the U21 international team. Six players had been selected in both younger and the senior international teams, but not in U21; these players were not included in the comparison between the three aforementioned groups, but were included in the retrospective analysis of senior international players.

### Data collection procedures

The data on national team appearances was collected from the Swedish Football Association’s website (https://www.svenskfotboll.se/landslag/). This included manually searching for the identified players using the website’s search function. For several players, the data of junior clubs was also checked on transfermarkt.com. The first data was searched and collected in December 2022. This was supplemented by data at club level and new national team data in December 2023. The accuracy and completeness of the data was finally checked in June 2024. The variables collected and their definitions are outlined in Table 1. Since this project did not collect any health-related data, did not involve any physical intervention on a subject, did not use any method intended to affect the subject physically or psychologically, and did not process any sensitive personal data, it did not fall under the Swedish Ethical Review Act and therefore did not need to be approved by an ethics committee.

**Table 1:** Variables of interest and accompanying definitions.

Variable	Definition
Youth international team debut	The international age group squad at which each player was first selected
Early pathway	International debut at U15–U16
Middle pathway	International debut at U17–U19
Late pathway	International debut at U21 or senior
Senior international debut	Age at which the player made their first official appearance for the senior international team, excluding friendly fixtures
Premier division debut	The age at which each player made their first official appearance for a premier division club or international club, where appropriate
Age of elite club entry	Age at which the player was first recruited into a club at international, premier or second division level.
Junior club level	Level of the club where the player played aged 16–18 years; premier, second, third division, or international club

### Statistical analysis

The descriptive statistics for each group are presented as mean and standard deviation (M ±  SD) for continuous variables and as frequencies with percentages for count variables. To compare the player groups, the differences between the groups were reported with a 95% confidence interval (CI) for continuous variables. For categorical variables, chi-square tests using a Monte Carlo simulation with 2000 replicates were used, followed by a standardized residuals table. An alpha level of 0.05 was used. This means that a 95% CI that does not exceed 0 indicates significant differences. Standardized residuals greater than 1.96 indicate significant over- or under-representation in a category. All analyses were performed in R 4.4.1.

## Results

### Club environment

Comparing premier division debut ages between U21 and senior international players (Table 2), senior only players had a later club premier division debut than U21 only (-2.3 years, 95% CI [-3.7, -0.9 years] and both U21 and senior (-2.0 years, 95% CI [-3.4, -0.6 years] players. There were no differences in the age of senior international debut or number of senior international matches.

**Table 2:** Debut ages and number of matches of U21 and senior national team players.

	U21 only (n = 195)	U21 and senior (n = 98)	Senior only (n = 14)	U21 only – U21 and senior		U21 only – Senior only		U21 and senior – Senior only	
	Diff	95% CI	Diff	95% CI	Diff	95% CI
Senior NT debut (years)	—	25.1 ± 3.5	26.9 ± 3.3	—	—	—	—	-1.8	[-3.8, 0.2]
Senior NT matches (n)	—	24 ± 24	12 ± 18	—	—	—	—	11	[-0, 23]
Club premier division debut (years)	19.3 ± 1.8	19.5 ± 1.5	21.5 ± 5.1	-0.2	[-0.6, 0.2]	-2.3	[-3.7, -0.9]	-2.0	[-3.4, -0.6]
Age of elite club entry (years)	13.6 ± 4.0	14.6 ± 3.8	15.5 ± 5.1	-1.0	[-2.0, 0.0]	-1.9	[-5.2, 1.4]	-0.9	[-4.2, 2.4]

(Mean ±  standard deviation).

**Fig 1:**
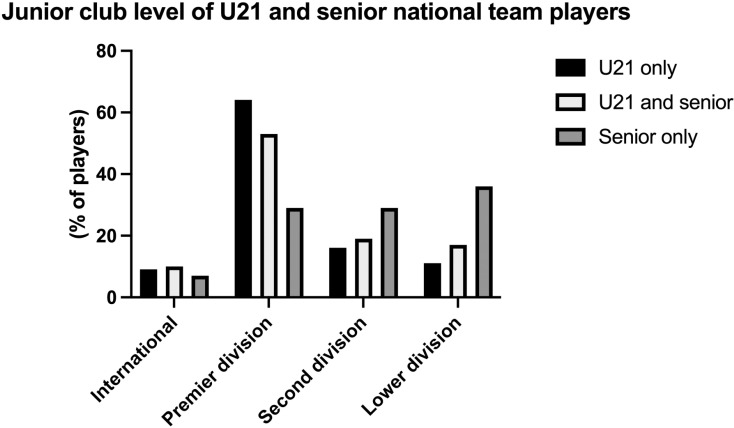
Junior club level (age 16–18) of U21 and senior national team players.

The junior club level of U21 and senior players is shown in Fig 1. There was no significant difference in level between U21 only, U21 and senior, and senior only players (□^2^ =  12.1, *p* = .063). However, the standardized residuals indicates an over representation of U21 only players from premier division clubs, and under representation of players in lower division clubs. In the senior only group, there was an under representation of players from premier division clubs, and an over representation from lower division clubs (Table 3).

**Table 3:** Junior (age 16–18) club level of U21 and senior national team players.

	Observed frequencies n (%)			Diff from expected frequencies n (standardized residuals)		
Junior club level	U21 only	U21 and senior	Senior only	U21 only	U21 and senior	Senior only
International	18 (9%)	10 (10%)	1 (7%)	0 (0.10)	1 (0.25)	0 (0.32)
Premier division	121 (64%)	52 (53%)	4 (29%)	10 (2.33)	-5 (1.36)	-4 (2.33)
Second division	31 (16%)	19 (19%)	4 (29%)	-3 (0.92)	1 (0.47)	1 (0.92)
Lower division	20 (11%)	17 (17%)	5 (36%)	-6 (2.21)	3 (1.20)	3 (2.21)

Difference from expected frequencies was calculated as: Observed frequency – expected frequency. Standardized residuals > 1.96 indicate a significant over or under representation.

### Senior international team players

**Fig 2:**
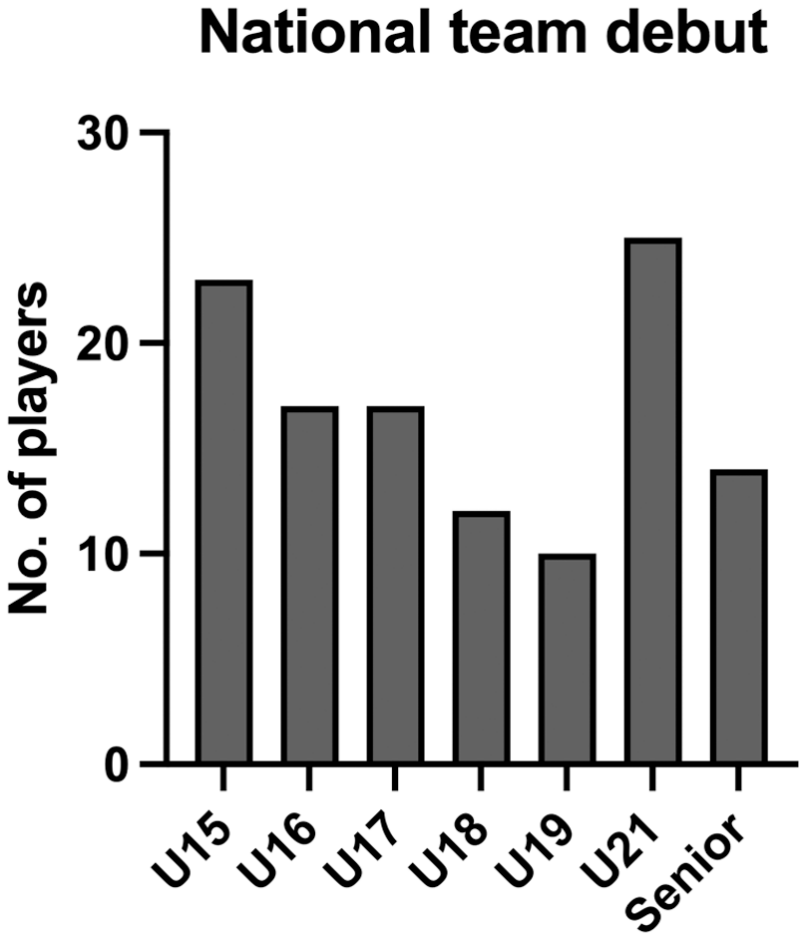
National team debut category of senior national team players.

The senior international debut age was 19–34 years, on average 25.3 ±  3.5 years. The number of senior international matches ranged between 1–93, on average 22 ±  24 (Table 2). The youth international team debut for the senior international players can be seen in Fig 2. The premier division debut ranged 17–26 years, on average 19.8 ±  1.8 years. The age of elite club entry was 4–20 years, on average 14.4 ±  4.1 years. The junior club level was international (n =  12; 10%), premier division (n =  60; 51%), second division (n =  24; 20%), and lower divisions (n =  22; 19%) (Table 2).

### Player pathways

Of the 118 senior international players, 40 (34%) had and early pathway (debut in U15–16 national teams), 39 (33%) had a middle pathway (debut in U17–19 national teams), and 39 (33%) had a late pathway (debut in U21 or senior national teams). Comparing debut ages between the different pathways, late pathway players had a later senior international debut than early pathway players (-2.5 years, 95% CI [-4.0, -1.0 years]). Late pathway players also made their senior club debut later than middle (-1.3 years, 95% CI [-2.0, -0.5 years]) and early (-1.9 years, 95% CI [-2.6, -1.1 years]) pathway players. There were no differences in number of senior international matches or the age of youth club selection. There was no difference in youth international team debut between U21 only players and U21 and senior players (𝜒2 =  7.9, p = .015).

**Fig 3:**
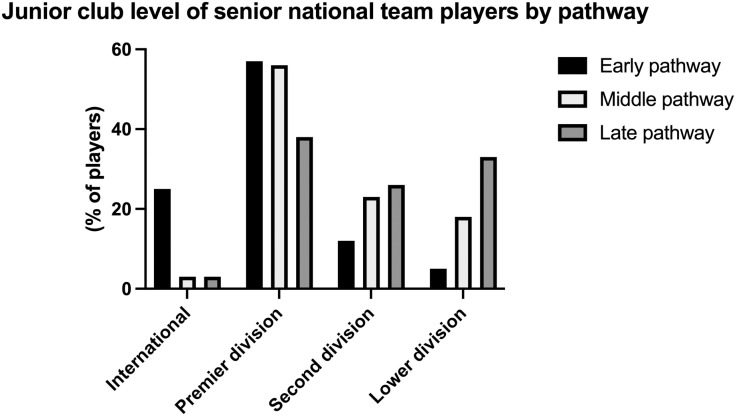
Junior club level (age 16–18) of senior national team players, by national team pathway.

The junior club level of players with different international pathways is shown in Fig 3. There was a difference in level between the different pathways (𝜒^2^ =  25.3, *p* = .001). In the early pathway, there was an over representation of players in international clubs, and under representation of players in lower division clubs. In the late pathway, there was an overrepresentation of players in lower division clubs.

## Discussion

This study examined the pathways of senior international footballers over 13 years by specifically exploring their accumulated participation at the junior international level and within their respective football clubs. Our analysis demonstrates that the pathways of the senior international players were highly individualised, with a relatively even balance of early, middle and late pathways. Whilst junior international participation was in no way a prerequisite for senior international selection, the significant majority of senior international players accumulated some junior international experience prior to senior representation. The age at which senior international players were first recruited into elite club environments varied, although the majority (60%) entered an international or Swedish premier division club at some point during their junior years.

The age at which young athletes are selected into formalised TD programmes has long been a topic of significant debate [3,8,10,12,13,15]. Indeed, given that the vast majority of successful junior athletes do not become successful senior athletes [8], there have been calls to delay the age at which athletes are recruited into such programmes and to widen developmental opportunities to broader populations [1,13]. Whilst Güllich and colleagues reported that ‘successful junior athletes and successful senior athletes are largely two disparate populations’ [8], our analysis show that 33% of senior international players were selected for the Swedish U15 or U16 international teams (Fig 1). These findings are interesting when compared to those of Söderström and Garn [21], also in the Swedish Football Association context, whereby players selected into regional TD programmes at U15 had a higher likelihood of progressing to professional football. On the other hand, our analysis also demonstrated that 40% of senior internationals developed in clubs lower than the highest/premier level.

Of the senior international players, there was an underrepresentation of players from premier clubs and an overrepresentation from second division clubs (Fig 2). In turn, players from lower ranked clubs are overrepresented among senior international players, whilst players from higher ranked clubs are overrepresented in those who only participated at U21 but not at the senior international level. However, it should be noted that in absolute terms, of all players who made an U21 *and* senior international appearance, the significant majority (72%) entered a premier or first division club at some point during their junior years (53% and 19%, respectively). Thus, the highest ranked clubs provided the greatest proportion of senior international players. Whilst the conversion rates of athletes from the junior to senior level are typically low across the literature [3,8,12,17], of those very select few who did reach the senior international level in our sample, the majority participated in a premier division club from 16–18 years of age. This is by no means surprising, as the relatively highest-performing players are usually recruited into the higher-ranked clubs.

The reason why the majority of senior international players were developed, at some point during their junior years, in the highest ranked club environments is probably attributable to a multitude of potential explanations. Factors including active player recruitment, invested resources, quality of facilities, levels of competitive challenge, number and quality of coaches, and the contact hours that players are exposed to will inevitably have some influence on their developmental trajectories as they progress through adolescence, and this developmental provision will differ between club environments [4,27–30]. Indeed, the Swedish academies today have a certification system similar to the Elite Player Performance Plan in the UK. In this system, football academies are ranked best upon their quality of developmental provision, with the higher ranked clubs being those that are perceived to be higher performing in a number of factors including productivity rates, training facilities, coaching, and education [31]. In Sweden, the higher ranked club environments are typically those with greater resources, facilities, coaches, and training contact hours. However, the club development environments in Sweden are notably different to those in the United Kingdom and the other leading football nations, with traditionally later recruitment processes in Sweden, lower number of professional football clubs, less full time academy coaches, and lower financial resources [32]. This notwithstanding, in our data, players from lower ranked club environments were overrepresented in the sample of senior international players. This illustrates that whilst access to higher ranked club environments during youth can be a feature that positively influences development, it is not essential for senior international representation. Further, the overrepresentation of senior international players from lower ranked club environments also demonstrates the need to consider the complexity of biopsychosocial factors that influence development and not just the age at which players are selected, the ranking of their club environment, or their formalised training hours [4,33].

Focussing specifically on senior international players, the significant majority accumulated junior international experience prior to making senior international appearances. One in five senior international players were recruited into the international pathway at the U15 level. After the U15 age group, progressively fewer senior international players were recruited at the U16–U19 level, spanning from 14–8% (Fig 1). This can be partly explained by the fact that there is no broad selection pyramid in these years, as there is before the first national team at the age of 15. It may therefore be less likely for players outside the system to be selected in these years. At the U21 level, the percentage rises again to a similar proportion of players who were recruited at U15. Essentially, our data shows that approximately an equal number of senior international players are recruited into the international pathway at the U15 group as those at the U21 group. This is supported by the data in Fig 3, whereby nearly the same number of senior international players had an early pathway compared to those that had a late pathway. Yet, interestingly, more senior international players made their youth international debut for the younger international teams (U15, U16 and U17; 47%) than those for the older youth international teams (U18, U19, U21; 39%). In our analysis, it was relatively rare that senior international players did not accumulate any junior international experience prior to their senior international debut (n =  14, 12% of the population).

Combined, these findings pose several implications from a TD perspective. What is clear is that these players’ pathways were highly individualised, dynamic and non-linear in nature [7]. This is evidenced by the fact that just as many senior international players were recruited into the international pathway at the first junior international age group (U15) as those who were recruited at the last junior age group (U21). Moreover, there is a relatively even spread of senior international players who had an early, middle or late pathway. Some players were selected at the very first international age group, participated in every subsequent age group, and made senior international appearances; others were selected at the first age group, were deselected and then re-selected several years later, and then made a senior international appearance. Other players made no junior international appearances prior to senior international representation. These individualised and non-linear pathways are reminiscent of those outlined by high-performing athletes in alternate contexts [10,24,30,34–36].

Secondly, whilst the low conversion of youth athletes to the senior professional level are often used as evidence to suggest the re-direction of developmental resources away from the very select minority toward broader populations [13], our data shows that junior international participation is relatively frequent in senior international players. Data from alternate contexts suggests that the challenges and experiences provided in these formalised TD environments during youth can be important in shaping those who do ‘make it’ to the elite senior level [24,30]. However, it is not known whether the senior international players with prior international youth experience would have taken a different development path without such development opportunities and experiences. It is also important to recognize that as the base of the TD pyramid narrows, the majority of elite players will naturally emerge from these formalized pathways. This self-reinforcing process should caution us against judging the success of the system only by the number of players who pass through these pathways, without considering other potential development pathways. Furthermore, those selected for premier clubs and junior international teams represent a minute fraction of players in their respective age groups [9,10], meaning that a significant majority are also denied such developmental provision [13], particularly those who are not subject to a range of early advantages [14,37–45]. Some of these non-selected players may too have had different developmental trajectories if provided such opportunities. Our data, therefore, highlights the importance of a coherent TD system across an Association, with multiple entry, exit, and re-entry routes and with collaboration between age groups, clubs, and the Association itself [4,46–52]. This is evidenced by the even spread of early, middle and late pathways in our data, whereby players had multiple opportunities to bounce in and out of multiple TD systems throughout early adolescence until adulthood to support their pathway to the senior international level. As such, an important implication from this work is that all routes to the senior professional should remain as accessible as possible (within practical, financial, and resource constraints) to ensure that the talent pool is as broad as possible.

We acknowledge that we analysed 313 male players that played in the senior or U21 international team between 2011–2023 from just one Football Association. Thus, the findings are specific to Swedish male international football and caution is encouraged when drawing direct implications to alternate contexts. Secondly, players who participated at the junior international level but not at the U21 or senior international level were not included. Thus, this is an analysis of those who did ‘make it’ rather than those who did not ‘make it’ [53]. Moreover, we are unable to provide any specific detail regarding the qualitative and quantitative differences between the different rankings of the respective club levels (e.g., between premier, first division, second division clubs). The inclusion of such factors present important directions for future research.

## Conclusions

Our findings show that the pathways to the senior international level are dynamic, non-linear, and highly individualised. Senior international players were recruited at the club and international level at early stages *and* at late stages of adolescence, and also anywhere in-between. Whilst youth entry into elite club environments and junior international teams is in no way a prerequisite for senior success, it does make it more likely, and youth players within the highest ranking clubs progressed and represented the greatest proportion of senior international players in our data. Interestingly, however, players from lower ranked clubs were overrepresented among the players who reached the senior international team without previous youth international team experience, and made a later debut in the senior international team. We show that international senior players take different paths to the top and encourage clubs and associations to create coherent and integrated structures that allow players to enter and leave different development environments that best suit their individual needs during their journey to the senior level.

## Supporting information

S1 Table Outcome variables. Collected data variables with the raw data.
(XLSX)
